# Monitoring the Neuroinflammatory Response Following Acute Brain Injury

**DOI:** 10.3389/fneur.2017.00351

**Published:** 2017-07-20

**Authors:** Eric Peter Thelin, Tamara Tajsic, Frederick Adam Zeiler, David K. Menon, Peter J. A. Hutchinson, Keri L. H. Carpenter, Maria Cristina Morganti-Kossmann, Adel Helmy

**Affiliations:** ^1^Division of Neurosurgery, Department of Clinical Neurosciences, University of Cambridge, Cambridge, United Kingdom; ^2^Department of Clinical Neuroscience, Karolinska Institutet, Stockholm, Sweden; ^3^Division of Anaesthesia, Department of Medicine, University of Cambridge, Cambridge, United Kingdom; ^4^Rady Faculty of Health Sciences, Department of Surgery, University of Manitoba, Winnipeg, MB, Canada; ^5^Clinician Investigator Program, Rady Faculty of Health Sciences, University of Manitoba, Winnipeg, MB, Canada; ^6^Wolfson Brain Imaging Centre, Department of Clinical Neurosciences, University of Cambridge, Cambridge, United Kingdom; ^7^Department of Epidemiology and Preventive Medicine, Monash University, Melbourne, VIC, Australia; ^8^Department of Child Health, Barrow Neurological Institute at Phoenix Children’s Hospital, University of Arizona College of Medicine, Phoenix, Phoenix, AZ, United States

**Keywords:** neuroinflammation, traumatic brain injury, subarachnoid hemorrhage, multimodal monitoring, secondary brain injury

## Abstract

Traumatic brain injury (TBI) and subarachnoid hemorrhage (SAH) are major contributors to morbidity and mortality. Following the initial insult, patients may deteriorate due to secondary brain damage. The underlying molecular and cellular cascades incorporate components of the innate immune system. There are different approaches to assess and monitor cerebral inflammation in the neuro intensive care unit. The aim of this narrative review is to describe techniques to monitor inflammatory activity in patients with TBI and SAH in the acute setting. The analysis of pro- and anti-inflammatory cytokines in compartments of the central nervous system (CNS), including the cerebrospinal fluid and the extracellular fluid, represent the most common approaches to monitor surrogate markers of cerebral inflammatory activity. Each of these compartments has a distinct biology that reflects local processes and the cross-talk between systemic and CNS inflammation. Cytokines have been correlated to outcomes as well as ongoing, secondary injury progression. Alongside the dynamic, focal assay of humoral mediators, imaging, through positron emission tomography, can provide a global *in vivo* measurement of inflammatory cell activity, which reveals long-lasting processes following the initial injury. Compared to the innate immune system activated acutely after brain injury, the adaptive immune system is likely to play a greater role in the chronic phase as evidenced by T-cell-mediated autoreactivity toward brain-specific proteins. The most difficult aspect of assessing neuroinflammation is to determine whether the processes monitored are harmful or beneficial to the brain as accumulating data indicate a dual role for these inflammatory cascades following injury. In summary, the inflammatory component of the complex injury cascade following brain injury may be monitored using different modalities. Using a multimodal monitoring approach can potentially aid in the development of therapeutics targeting different aspects of the inflammatory cascade and improve the outcome following TBI and SAH.

## Introduction

### Pathophysiology of Brain Injury

Traumatic brain injury (TBI) and aneurysmal subarachnoid hemorrhage (SAH) are common neurological conditions (1, 2) associated with extensive morbidity and mortality (3, 4). While the two diseases are different entities, they share many common features in their secondary pathophysiology. Furthermore, they provide an experimental paradigm in which multimodality monitoring offers the opportunity to decipher common pathological processes. There are multiple potential mechanisms by which neuronal injury is inflicted following TBI and SAH. The traditional clinical paradigm for management of these conditions has focused on adequate delivery of oxygen and appropriate metabolic substrates to the injured brain, and prevention of secondary injuries caused by hypotension and hypoxia (5). These secondary processes lead to an increasingly inhospitable environment with ongoing excitotoxicity, oxidative stress, blood–brain barrier (BBB) disruption, cortical spreading depression, mitochondrial dysfunction, and subsequent cellular death in the tissue surrounding the initial damage (6, 7). There is an increasing recognition that inflammatory mediators are involved in the mechanistic link that underlies these injurious processes (8–10).

### What Is Neuroinflammation?

**Figure 1:**
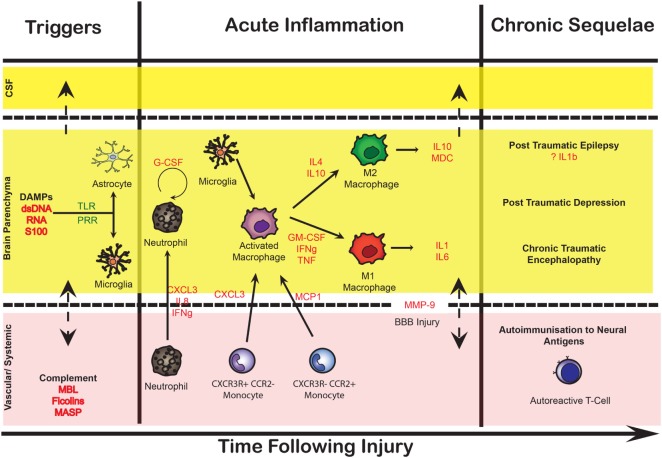
Conceptual summary of the neuroinflammatory response in acute brain injury. This illustration depicts some of the cellular and molecular sequelae and inflammatory cascades that occur following injury to the brain over time, divided into different compartments [cerebrospinal fluid (CSF), brain parenchyma, and vascular]. Initially, danger-associated molecular patterns (DAMPs) including double-stranded (ds)DNA, RNA, and brain-enriched proteins such as S100B provide early triggers to resident cells reflecting tissue injury and linking the initial insult to subsequent humoral and cellular cascades. These in turn lead to an increasingly cytotoxic environment promoting cellular responses, stimulating both immunocompetent cells in the central nervous system (resident microglia and astrocytes), as well as recruiting peripheral monocytes, through pattern recognition receptors (PRR) such as toll-like receptors (TLR). The secretion of cytokines, chemokines, and other potent chemotactic substances (including complement proteins) induces the recruitment of neutrophils and monocytes into the brain parenchyma through the increasingly permeable blood–brain barrier (BBB), as well as having a direct cytotoxic effect. Ongoing release of cytokines initiates a long-term inflammatory process, which allows the dynamic shift of macrophages and microglial canonical phenotype between M1 (classical activation) and M2 (alternative active presumably the result of antigen-presenting cells migrating from the periphery). These processes result in chronic inflammation as well as auto-immunization toward brain-enriched antigens. Key cytokines are highlighted in red. MBL, mannose-binding lectin; MASP, mannose-associated serine protease; MMP-9, matrix metalloproteinase 9.

Galen’s original description of inflammation as *calor* (heat), *dolor* (pain), *rubor* (redness), *tumor* (swelling), and subsequently *functio laesa* (loss of function) has been refined into a more complex phenomenon that represents the host response to any insult. This complex process involves the release of molecular mediators, the alteration of the cerebral vasculature, the activation and influx of immune cells to eliminate pathogens, damaged cells, and other perceived harmful stimuli. The brain was long thought to be immunoprivileged due to the presence of the BBB, which limits the cross-talk between the blood and brain-resident inflammatory cells, the lack of a classical lymphatic system, and the shielding of neural antigens from peripheral immune surveillance. More recently, all these assumptions have been questioned and revised (11). There is an increasing realization that cerebral inflammation or neuroinflammation occurs within the whole gamut of central nervous system (CNS) pathologies, whether it is an adaptive autoimmune response (e.g., multiple sclerosis) or a response to external stimuli (e.g., accumulation of red blood cells following brain hemorrhage). While microglia are considered the primary immune cells of the CNS, it is becoming increasingly clear that other glial cells (astrocytes and oligodendrocytes) as well as neurons display immune-competent functions. Activated resident cells of the CNS, in combination with migrating inflammatory cells from peripheral blood, form an intricate immune network (12, 13). Although this immune response may be initiated to protect the brain, it is becoming evident that it can result in harmful outcomes for the CNS (14). In fact, an ongoing neuroinflammatory response may not only contribute to increased edema, cellular death, and BBB disruption but also function as a potent scavenger of dead cells and support the regenerative processes in the injured CNS (15, 16). An outline of the pathophysiological mechanisms identified in the literature is illustrated in Figure 1, many of which are described in this review.

### Aspects of Neuroinflammation in SAH

**Figure 2:**
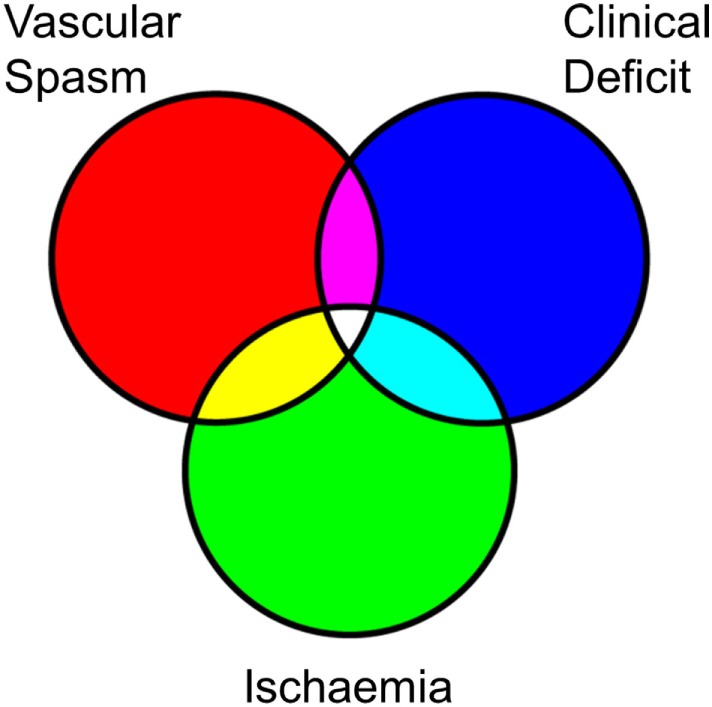
Overlapping characteristics of the pathophysiology in subarachnoid hemorrhage (SAH). Simplified Venn diagram of the different pathophysiological processes that follow SAH. Rather than a linear relationship between vascular spasm, ischemia, and clinical deficit, these may be overlapping and also occur as distinct phenomena. Vascular spasm is most closely linked with endothelin-1-mediated vasoconstriction but may also encompass swelling of the vascular endothelium leading to an angiographic appearance of reduced contrast flow. Ischemia may result from an absolute reduction in blood flow, which may be complicated by local microvascular collapse or thrombosis, or even hyperemia causing localized swelling and reducing the capillary perfusion in that locality. Neurological deficits may be caused by frank ischemia and also result from phenomena such as cortical spreading depression.

Brain hemorrhage triggers a series of pathological processes resulting in neuronal damage and consequent neurological deficit (17–22). Early brain injury (EBI) refers to the damage ensuing in the first 72 h after the bleed caused by transient cerebral ischemia (CI), BBB disruption, edema, cell death, and brain tissue loss. Many of the patients who survive these phenomena deteriorate days later from delayed ischemic neurological deficit (DIND), which is responsible for poor outcomes or death in up to 30% of patients with SAH. DIND was traditionally attributed to narrowing of the large basal cerebral arteries, termed cerebral or angiographic vasospasm, causing a reduction in cerebral blood flow (CBF) and, if critically reduced, progressing to infarction in the relevant vascular territory. However, a growing number of reports shows that angiographic vasospasm does not always correlate with CI, cerebral infarction, or changes in CBF, indicating that DIND is a more complex (and complicated) phenomenon (23–33). The landmark CONSCIOUS-1 trial showed that an endothelin-1 antagonist ameliorates angiographic vasospasm, but fails to improve functional outcomes (34, 35), forever changing the concept of DIND, which is now suspected to arise from the combined effects of angiographic vasospasm, global reduction in blood flow, arteriolar constriction and thrombosis, cortical spreading depressions, and processes triggered by EBI (17–21, 36). With the complex interplay of the underlying pathological substrates, DIND is often (incorrectly) used as a blanket term for vascular spasm, CI, and neurological (clinical) deficit. It remains unclear to what extent these phenomena overlap or represent distinct processes (Figure 2).

It is increasingly recognized that the neuroinflammatory cascade following SAH is the potential mechanistic link between the initial ictus and the progression of neuronal injury in EBI as well as the development of DIND (37–40). Both overlapping phenomena of ischemic injury and vasospasm lead to an unfavorable physiological environment that may inflict neuronal injury (Figure 2), possibly driven in part by neuroinflammatory mediators.

### Monitoring Neuroinflammation in the Neuro-Critical Care Unit (NCCU)

Patients suffering from TBI and SAH are typically treated in specialized NCCUs where extensive intracranial monitoring is implemented in order to detect subsequent secondary insults, prevent further deterioration, and optimize conditions for brain recovery (41, 42). Monitoring neuroinflammatory processes is technically and logistically complex, and this has hampered the development of pharmaceutical compounds that act to modulate the neuroinflammatory cascade (43). As we acquire a greater insight into the interplay between neuroinflammation and secondary brain injury, monitoring the neuroinflammatory events may open an opportunity to both increase our understanding of the molecular basis of the underlying pathology as well as identify novel therapeutic targets.

The aim of this narrative review is to demonstrate how different aspects of the neuroinflammatory response contribute to the pathophysiology of secondary brain injury. In addition, we discuss the methods available to monitor cellular and humoral inflammation in TBI and SAH patients in the clinical setting.

## Cytokine and Chemokine Monitoring in Acute Brain Injury

There is no accepted gold standard in how to assess the neuroinflammatory processes in TBI and SAH. In clinical studies, the most common approach is to measure the concentration of cytokines and chemokines as biomarkers of ongoing neuroinflammation. Cytokines are small proteins of approximately 20 kDa molecular weight that are synthesized primarily by immune cells to promote and regulate inflammatory responses in an autocrine and paracrine fashion.

There are currently a wide range of commercially available assays that can detect with high sensitivity (pg/ml range) the levels of cytokines in different compartments. Historically, enzyme-linked immunosorbant assay has most frequently been used, but these require relatively large volumes of fluid/tissue and can only measure one cytokine at the time, and are therefore not ideal to monitor a complex inflammatory network. In the last 10 years, multiplex bead arrays have allowed the use of very small sample sizes (~25 μl per sample) to simultaneously measure several cytokines, thus greatly increasing the technical efficiency and our understanding of the inflammatory response in the injured brain (44).

### CNS Compartmentalization of Cytokine Measurements

The CNS has several inherent barriers, restricting and controlling the flow of substances. These include the BBB, the blood–cerebrospinal fluid (CSF) barrier, and the CSF–brain barrier. The brain compartment, or specifically the extracellular space of the brain, is accessible by using a microdialysis (MD) catheter. The MD catheter possesses a semipermeable membrane that is able to extract proteins like cytokines directly from the extracellular fluid (ECF) when inserted into the injured brain (13, 45).

The CSF is produced by cells of the choroid plexus located within the cerebral ventricles and provides a medium to embed the brain, maintain a homeostatic environment, and facilitate the transport of nutrients and waste products. Thus, while using the CSF for cytokine measurement may provide a global assessment of cerebral inflammation, it remains less specific in terms of regional monitoring. CSF is more easily accessible through, ideally, external ventricular drainage in sedated patients or *via* lumbar puncture in conscious patients where several milliliters can be drawn for analyses. While blood is biologically the most remote space from the organ of interest, it represents an easily accessible compartment for repeated sampling. To compare and monitor cytokine concentrations, collection of matched CSF and plasma samples over time allows a composite picture of ongoing immune responses, distinguishing central from systemic cytokine profiles and determining how immune activation in the brain may impact on mechanisms occurring in the periphery and *vice versa*.

Figure 1 illustrates the intricate relationship between different compartments in the CNS and the potential cross-talk between the different compartments.

### Cytokines in Blood

Although controversial, some groups have correlated cytokine concentrations measured in blood/plasma in the acute and chronic setting of patients with TBI and SAH to functional outcome (46–48). This suggests that, while these mediators may represent non-brain-specific inflammatory activity, systemic inflammation is triggered by brain injury, potentially having long-term sequelae such as stimulating an acute-phase response in the liver (49). Importantly, the pattern of cytokine production is both qualitatively and quantitatively different in blood compared to CSF following injury (49–52), suggesting the existence of distinct biological processes at play.

### Cytokines in CSF

Collection of CSF is the oldest and most prevalent method to test for inflammatory mediators following TBI and SAH and, and in contrast to blood sampling, it represents a global measure of cerebral immune activity. Access to ventricular CSF is achieved with surgical insertion of a drain in the ventricles to measure and monitor changes of intracranial pressure. Longitudinal measurement of cytokines in CSF of patients with severe TBI reveals a biphasic immune response being maximal in the first few days after injury followed by a second modest increase by 7–10 days as shown for IL-6 (49).

These data have been confirmed using principal component analysis (PCA) of CSF cytokines and chemokines, resulting in the identification of distinct clusters of cytokine response (53, 54). Interestingly, multivariate analysis has also demonstrated a significant difference in outcome between the two clusters. Thus, assessing inflammatory activity provides a potential window for appropriate pharmaceutical therapies targeting cytokine production (54). The difficulty is in disentangling the roles of individual mediators within these clusters to investigate rationalized treatment targets.

In SAH, CSF samples may not be a true measure of neuroinflammation. The volume of blood present within the subarachnoid space can affect the levels of cytokines present in CSF, and therefore, cytokine concentration in CSF is partly a consequence of the volume of SAH rather than a specific measure of the magnitude of the inflammatory response within the brain.

### Microdialysate Monitoring of Brain ECF

Microdialysis is inevitably a spatially restricted approach that can be used to target pericontusional/infarcted tissue at risk for secondary injury and thus allow us to monitor ongoing or worsening focal brain damage. Notably, cytokine analysis revealed large variations in their concentrations when comparing CSF with brain-ECF (Table 1). However, the difference in cytokine levels between CSF and brain-ECF is smaller compared to the concentration gradients between these compartments and serum (55). Measurement of cytokines using MD have the additional complication of relative recovery. Relative recovery within the microdialysate fluid refers to the recovered fraction of the true concentration in the ECF, which is governed by a range of physicochemical factors including the molecular weight of the cytokine, its isoelectric point (which in turn determines the charge the protein carries at neutral pH and its solubility in water), and its native oligomerization characteristics (56). For some species, the relative recovery can be as low as 5% (exemplified in Table 1) making it technically difficult to reliably monitor.

**Table 1:** Comparison of selected cytokine concentrations in serum, CSF, and microdialysate in patients with traumatic brain injury.

Cytokine	Mean recovered MD concentration from ECF (pg/ml)	RR (%)	Mean recovered ECF concentration, adjusted for RR	Mean CSF concentration (pg/ml)	Mean serum concentration (pg/ml)
IL-1β	10.4–20.8	~30%	34.6–69.3	9.9–89	0.5–7.4
IL-1ra	2,796	~30%	9,320	26,861	10–221
VEGF	200.1	~4%	5,003	26–43	37–773

*Concentrations of interleukin-1 beta (IL-1β), interleukin-1 receptor antagonist (IL-1ra) and vascular endothelial growth factor (VEGF) in cerebrospinal fluid (CSF), serum and microdialysate (MD) from extracellular fluid (ECF). The relative recovery (RR, in %) is noted and adjusted for. Data from Ref. (56, 58)*.

There is an intricate interplay between the various cellular elements (both resident and recruited) within the brain, which are both the source and target of these mediators (13, 57, 58), further complicating the interpretation.

Using either a qualitative approach or an unbiased quantitative analysis (such as PCA), cytokine production appears as an intrinsic and ordered component among the multiple biochemical pathways contributing to delayed brain damage (57, 59). This fact would argue against the inflammatory response being a secondary form of injury *per se*, and rather a sequential cascade of molecular events [in the words of Julius Caesar: *alea iacta est* (the die is cast)], with a predetermined sequence following the initial injury.

### Perfusate for Cytokine Recovery Using Microdialysis

MD is commonly used in the NCCU for metabolic monitoring of the injured brain, recovering small metabolites such as lactate, pyruvate, glucose and glycerol from the ECF (45). For these metabolites, the relative recovery is circa 70% from catheters inserted into the brain, using a flow rate of 0.3 µl/min (60). Importantly, the pore size of MD membranes used for metabolite analysis is significantly smaller (20 kDa) compared to the size used for larger proteins such as cytokines (100 kDa) (61, 62). By using 100 kDa cutoff catheters with the standard crystalloid solution, the relative recovery for cytokines has been shown to be typically 30%, but as low as 5% for cytokines that oligomerize (e.g., TNF) (56). In order to improve poor recovery rates, colloids such as human albumin solution (HAS) or dextran may be added to the perfusion fluid (63). The addition of colloids does not always improve the recovery of all cytokines; however, the relative recovery for TNF in 3.5% HAS has been shown to increase from 4.4 to 31.2%, a sevenfold increase (56). A 3% Dextran-500 solution shows equally encouraging results compared to crystalloid solutions (64), and has the capacity to improve extraction of even larger inflammatory mediators (65, 66) and has been empirically demonstrated to remain within a 100 kDa membrane catheter (67). In summary, the optimal recovery of cytokines within the MD samples is dependent upon perfusate supplemented with albumin or dextran, favoring an improved relative recovery and no net loss of fluid from the catheter. In our opinion, this is an essential adjunct for protein microdialysis in general and specifically in recovering inflammatory mediators.

### Statistical Approaches to Cytokine Studies

While the neuroinflammatory cascade following TBI is complex and characterized by substantial collinearity and functional overlap among mediators, many studies have focused on single cytokines utilizing univariate comparative statistics without adequately assessing the interrelationships with other factors (68–70). A more appropriate statistical approach would be to incorporate several cytokines into multivariate analysis methods, such as PCA (71). By using PCA, it is possible to detect the greatest sources of variance in the dataset and thus highlight patterns in the cytokine response (59). A regression extension of PCA such as partial least square discriminant analysis (PLS-DA) can be used to identify variations in a particular domain such as time (58, 72). PLS-DA has the disadvantage of introducing bias, but can be used to test prespecified hypotheses.

### Interpretations of Cytokine Studies in TBI

A systematic review has identified 32 studies describing the analysis of CSF cytokines in 1,363 severe TBI patients, of which 19 studies reported the analysis of cytokines *via* MD in 267 severe TBI patients (73). The majority of the MD studies have been published from a few centers of excellence in TBI research, known for their work in the field of cerebral MD. From these studies, it is evident that MD catheter positioning plays a crucial role in the amount of cytokines recovered, with peri-lesional or lesional locations displaying greatly different cytokine profiles compared to “healthy” non-lesioned tissue. There is also a stereotyped response to injury whereby the peaks of MD derived cytokines such as interleukin-1 beta (IL-1β), IL-6, and IL-8 occur within the first 48–72 h post-TBI, with IL-10 remaining elevated throughout the acute period up to 5 days of monitoring. Therapeutic modulation of the neuroinflammatory response to TBI has been demonstrated clinically in patients using subcutaneous administration of recombinant human IL-1 receptor antagonist, with the aim of attenuating the experimentally proven IL-1-mediated cellular damage in the injured brain (57, 58). Several studies have attempted to correlate CSF cytokine concentrations [IL-1β, interleukin-1 receptor antagonist (IL-1ra), IL-6, IL-8, IL-10 and TNF] with patient outcome, demonstrating a number of conflicting data [summarized in a review article (74)]. We would argue that this approach is flawed as it ascribes a unitary role for a given cytokine (as “good” or “bad”) as well as ignoring the high degree of collinearity between all these mediators (75). It is important to recognize the delicate interplay between mediators such that the ultimate effect of a given cytokine is determined by the specific cellular, chemical, and temporal milieu in which the cytokine is acting. Finally, the clinical complication profile with brain-ECF and CSF-based cytokine sampling appears to be quite low, attesting to the safety of both procedures in critically ill TBI patients. All these findings are necessarily limited by the small number of studies identified in the accompanying systematic review.

### Interpretations of Cytokine Studies in SAH

Similar to the findings identified in the review on TBI, the studies reporting on the analysis of cytokines *via* MD and CSF in aneurysmal SAH cytokine can be found in the parent systematic review, included in this Research Topic in Frontiers in Neurology (Zeiler et al., 2017, II submitted). Overall, there are 9 studies describing the analysis of cytokines *via* MD in 246 aneurysmal SAH patients. Another 20 studies reported the analysis of CSF cytokines in 630 patients. As with the TBI cytokine literature, the MD and CSF studies identified within the review originated from a small number of centers of excellence in NCCU and SAH based research. This body of literature is smaller than that identified within the TBI population. Thus, the conclusions that can be made within the SAH systematic review are somewhat limited. However, as with the TBI literature, some interesting trends should be highlighted. Again, MD catheter location plays a role in the cytokine levels obtained in SAH patients, with lesioned tissue (i.e., ICH, ischemic brain) and peri-lesional tissue displaying higher cytokine levels than the “healthy” non-lesioned tissue. Second, SAH patients displayed peaks in MD IL-1β, IL-6, and IL-8 within the first 12 h’ post-hemorrhage, while (as in TBI) IL-10 appears to remain elevated within the acute period. Third, CSF-based cytokines in SAH appear to correlate with patient outcome, but as with the TBI data, our interpretation is that this type of solitary comparative analysis for each given cytokine may be misleading as there will be a potent collinearity between all these mediators. The association between cytokine profiles and the incidence of clinical vasospasm post-SAH has been documented in a previous systematic review and meta-analysis, with IL-6 and TNF linked to the development of vasospasm and delayed ischemic neurological deficit (DIND) (76). In addition, chronic hydrocephalus and shunt dependency may be associated with TGF-β levels within the CSF for SAH patients.

## Tissue Sampling to Assess Neuroinflammation

A direct method to determine focal inflammatory activity is to biopsy samples adjacent to the injured tissue. Compared to indirect sampling of fluid, this provides a measure of tissue inflammation with a high degree of confidence; however, it remains a temporal snapshot that cannot readily reflect dynamic processes. Harish et al. found that surgically removed tissue demonstrates higher concentrations of several, predominantly pro-inflammatory, cytokines, and chemokines in contused brain vs pericontusional tissue (77). They also found a high degree of macrophage and microglia activity in contused regions, suggesting strong inflammatory activity. Similarly, Bellander et al. identified increased complement activation from pericontusional tissue (78), indicating a strong inflammatory response, especially in surrounding neurons, following TBI. Additionally, in postmortem tissue of TBI patients, it has been shown that pro-inflammatory cytokine mRNA and protein concentration are significantly elevated compared to cytokines with a more anti-inflammatory function (79). In the same study, regions with a profound cytokine production were associated with abundant inflammatory cell infiltration, astrogliosis, and axonal pathology. While in postmortem tissue, changes that occur at the time of death might influence results, this study directly demonstrates that cytokines are upregulated in the brain parenchyma as early as a few minutes from brain injury. In the future, it might be possible to directly assess the inflammatory profile of the brain by acquiring brain tissue samples.

## Imaging Techniques to Measure the Neuroinflammatory Response

Advances in neuroimaging have allowed aspects of the neuroinflammatory cascade to be assessed with a high degree of spatial resolution. Ideally, these techniques can be combined with other tools such as MD, which are focal but provide temporal resolution, to get a more complete overview of inflammatory processes.

### Positron Emission Tomography (PET)

Positron emission tomography relies on radioligands, which bind to a specific receptor or mimic a biological molecule of interest, and can be used in *in vivo* studies of neuroinflammation (80). Translocator protein (TSPO) is a 18 kDa mitochondrial membrane protein involved in steroid biosynthesis and is by far the most studied target to quantify microglial activation following TBI (81, 82). The first-generation TSPO ligand, [^11^C]-PK11195 is the most commonly employed; however, there are now several second-generation agents with improved signal to noise ratio and reduced non-specific binding. The second-generation agents all demonstrate variable binding between individuals relating to a specific genetic polymorphism resulting in different TSPO affinity for the radiolabeled ligands (83). PET studies have revealed that the binding of TSPO ligand PK-11195 remains elevated chronically, many years following TBI (81, 82), and that the degree of binding, and therefore chronic microglial activation, correlates with the degree of cognitive impairment (81). The second-generation TSPO ligand [^11^C]DPA-713 has been used to demonstrate cellular neuroinflammation in retired American football players (84, 85).

Translocator protein ligands are limited by their overexpression in reactive astrocytes, complicating the interpretation of the resulting binding (86, 87). Moreover, expense and the ability to generate radioligands on-site in the acute phase make the logistics of scanning complex. Nevertheless, currently, TSPO PET remains the only method for human *in vivo* imaging of microglial activation.

Other putative PET ligands to assess neuroinflammation include cannabinoid-2-receptor (88), cyclo-oxygenase (COX) (89), and matrix metalloproteinases (90) but these have not been adequately studied in acute brain injury. Furthermore, it is also possible that (^18^F)-FDG-PET may be applicable to imaging neuroinflammation, because of the apparent linkage between metabolic programing and inflammation (see below).

### Magnetic Resonance Imaging (MRI)

The clinically used MRI sequences may be used to explore specific features of neuroinflammation (91), most commonly by assessing BBB integrity through the addition of the contrast agent gadolinium (92). In preclinical experimental settings, there are more advanced MRI applications, employing microparticles iron oxide, endothelial vascular cell adhesion molecule-1 (VCAM-1) (93), and macrophage-specific epitopes (94). However, in clinical studies in humans, MRI has not been employed to directly assess neuroinflammation, rather indirectly provide a measure of BBB dysfunction and endothelial cell activation.

### Magnetic Resonance Spectroscopy (MRS)

The prevalent form of *in vivo* spectroscopy is ^1^H magnetic resonance spectroscopy (^1^H-MRS, also called proton MRS). This is employed to assess relative levels of cerebral metabolites. MRS is also known as NMR spectroscopy, but usually the term MRS refers to *in vivo* measurements and NMR to *ex vivo* or *in vitro* measurements. The most abundant signal in the brain ^1^H spectrum is usually *N*-acetylaspartate (NAA). The peptide *N*-acetylaspartylglutamate (NAAG) is a product of NAA and the two are interconvertible. NAAG is a small peak in the brain ^1^H-MRS that is difficult to distinguish from NAA. Often both are considered together as “total NAA” that is interpreted as a marker for neuronal health, viability, and/or number of neurons, particularly their mitochondria. Reduction in NAA is regarded as indicating dysfunction (permanent or temporary) of neuronal tissue. Other ^1^H signals include creatine (combined signal from creatine and phosphocreatine), glutamate and glutamine (often considered together as Glx as the two species’ signals are incompletely resolved), gamma-aminobutyric acid, and lactate. ^1^H MRS also allows detection of elevated levels of myo-inositol (osmolyte present in glial cells) and choline containing compounds. Commonly, MRS is used to measure metabolic activity in TBI (95), but these substances have also been shown to act as a surrogate of neuroinflammation (91, 96).

Another form of MRS less commonly used is ^13^C-MRS, which necessitates administration (usually intravenously, or sometimes orally) of substrates such as glucose, acetate, lactate etc. that have been manufactured to be artificially enriched in ^13^C (e.g., 99%), in contrast to the natural abundance of ^13^C that is only 1.1% of all carbons. ^13^C-MRS methodology is a powerful means of quantifying metabolic fluxes *in vivo*, e.g., the tricarboxylic acid cycle and glutamate–glutamine cycling (97). Also, in recent years, ^13^C hyperpolarization has evolved, a technique that boosts the ^13^C-MRS signal albeit for a very brief duration. Clinically, it utilizes hyperpolarized 1-^13^C pyruvate, given intravenously, to measure glycolysis vs TCA cycle, by means of signals for the respective products 1-^13^C lactate and ^13^C HCO_3_^−^ (98). To date, it has mostly been used to image tumors (99), although results have emerged recently on ^13^C hyperpolarization in preclinical TBI (100, 101). ^13^C-MRS modalities may be useful in future in studies of neuroinflammation. A recent development (preclinically) is the use of hyperpolarized [6-^13^C] arginine to detect inflammatory cell function in cancer (102), and this could presumably be used in brain injury. A final MRS modality is ^31^P-MRS. No artificial enrichment is needed as ^31^P is virtually 100% of all naturally occurring phosphorus atoms. ^31^P-MRS is not commonly used, despite being able to measure energy-related phosphorus species non-invasively *in vivo*, including phosphocreatine, ATP, and inorganic phosphate. Notably, in a ^31^P-MRS study of TBI patients, Garnett et al. (103) suggested that the changes (vs healthy controls) might be due to reactive gliosis in the injured brain. The ^31^P-MRS modality is being actively investigated in acute brain injury.

## The Adaptive Immune System

In comparison to the innate immune system, the adaptive immune response has not been as extensively studied. It is believed that the transition between the innate and adaptive immune response following acute brain injury is moderated by migrating antigen-presenting dendritic cells (104).

### Cellular Components of the Adaptive Immune Response

The cellular components of the adaptive immune system can be measured in blood by identifying specific subpopulations of peripheral lymphocytes using flow cytometry. In severe TBI, this technology has revealed that the number of T-cells, specifically CD8^+^ cytotoxic cells, decrease significantly following TBI, while the number of B-cells remain constant (105).

### Autoimmune Responses to Neural Antigens

The humoral adaptive immune system is also activated following TBI as a result of presentation of previously novel neural antigens to the peripheral immune system. Acute brain injury triggers the production of autoantibodies toward brain-specific proteins, including the brain-enriched proteins glial fibrillary acidic protein (106) and S100B (107). Moreover, Cox and coworkers showed that peripheral blood mononuclear cells isolated from patients with TBI have the capacity to proliferate *in vitro* when stimulated by myelin basic protein, the most abundant protein of the myelin sheath expressed in the brain by oligodendrocytes (108).

These adaptive responses are long lasting and are a candidate mechanism in the later stages of the pathophysiology of brain injury that may underlie chronic neurodegeneration (109); however, the mechanism by which empirically identified autoantibodies inflict neuronal injury is yet to be proven.

## Failure of Clinical Trials Using “Anti-”Inflammatory Agents

There have been numerous failures to translate promising preclinical agents targeting the underlying conditions in brain injury studies into efficacious phase III human studies (110). Several reasons for this have been suggested, including small group sizes, inadequate dosing, inappropriate delivery route, inadequate therapy duration, and timing of drug delivery in relation to the occurrence of injury [several of the suggested harmful pro-inflammatory cytokines are elevated predominantly the first hours after injury (13)], the complexity of BBB and adequate drug penetration. In fact, large phase III studies, including corticosteroids (CRASH trial) (111), progesterone (112, 113), and erythropoietin (114) failed to detect the presence of the administered drug in the brain, something that could have been determined empirically using microdialysis (45). Moreover, due to poor penetration of pharmacological compounds across the BBB, lower concentrations of the drug may have reached the brain explaining the lack of expected neuroprotection (115). Before deciding to embark on large phase III ventures, which are costly and consume much time and resources, adequate phase II clinical studies with informative surrogate endpoints should be performed, including microdialysis, to ascertain the degree to which the drug can cross the BBB and exert changes on relevant biomarkers, including immune response-related molecules such as cytokines and chemokines.

## Conceptual Understanding of Mechanism of Action

Within recent years, there have been several ongoing trials aimed at negating the neuroinflammatory response in brain injury (43). As we improve the monitoring of the inflammatory response, we can improve the accuracy of prediction of how new anti-inflammatory drugs impact on the innate inflammatory response, and when they should be delivered, in order to best modulate neuroinflammation following brain injury. However, improved monitoring techniques also lead to increased study complexity as more data become available. Helmy and coworkers treated 10 patients with an IL-1ra (Anakinra) and they demonstrated that the cytokine response in ECF in the treated group resembled that of the classical pro-inflammatory microglial activation (M1) (116), with increased IL-1β and low levels of IL-4 and IL-10. These findings were counterintutitive given the classification of IL1ra as an “anti-inflammatory cytokine” and therefore more in keeping with the M2 (anti-inflammatory, adaptive) response that would have led to a predominance of anti-inflammatory cytokines (57). Nevertheless, the more data we obtain from the complex cascades in the neuroinflammatory response, the greater the refinement in our understanding. There is an increasing recognition that microglia and macrophages have the capability to express both M1 and M2 specific phenotypes (117), and that the classification system does not accurately reflect the complexity of microglial function (118).

Over recent years, the idea of metabolic reprogramming of macrophages associated with inflammatory phenotype has gained ground. Very recently, a study by Ip et al., in the context of inflammatory bowel disease, has shown that IL-10 opposes the switch to the more glycolytic metabolic program induced by inflammatory stimuli in macrophages (119). Specifically, IL-10 inhibits lipopolysaccharide-induced glucose uptake and glycolysis and promotes oxidative phosphorylation. These findings may be relevant to harnessing metabolic reprogramming to treat inflammation in injured brain.

## Chronic Sequelae of Neuroinflammation

Following brain injury, a chronic inflammatory state has been shown to correlate to several clinical conditions. One of the most common symptoms decreasing quality of life in patients that suffer from TBI is post-traumatic depression (PTD), which is present in up to 44% of patients (120). A study reported that higher levels of the acute CSF endothelial markers (sVCAM, sICAM-1, and sFAS) as well as IL-7 and IL-8, were associated with PTD as 6 and 12 months post-TBI, respectively (121).

Chronic traumatic encephalopathy (CTE) is a neurodegenerative condition leading to impairments in mood, behavior, cognition, and motor functioning and predominantly affecting patients with mild, repetitive TBI, common in contact sports (122). CTE has been defined as a tauopathy where phosphorylated tau accumulates within cells in the CNS (123). Recently, a cadaver study highlighted the presence of activated microglia (CD68-positive cells) close to tau deposits (124).

Another common complication following TBI is epilepsy (125), occurring in up to 20% of patients. Similarly, following SAH, a prevalence of between 7 and 25% has been reported (126, 127). Diamond et al. (128) found that a high CSF/plasma IL-1β ratio correlated with post-traumatic epilepsy (PTE) and that presence of a specific single-nucleotide peptide of the IL-1β gene (rs1143634) correlates with a higher risk of PTE following brain injury. Claassen and coworkers found an association between the inflammatory response following SAH (TNF-receptor 1 and high-sensitivity C-reactive protein) and the presence of seizures (129). Several reviews covering this field have been published, and all do suggest a strong link between neuroinflammatory cascades, increased excitotoxicity, and epileptogenesis in the aftermath of brain injury (130, 131).

It may therefore be possible to target inflammatory mediators after TBI and SAH, to alleviate the burden of these associated long-term morbidities.

## Collinearity and Confounding Factors Between Inflammatory Mediators

Collinearity is a statistical term that describes how two (or more) variables correlate with each other to a high degree. As most inflammatory mediators are present at a low concentration in the uninjured brain, and the subsequent increase in concentration is a response to injury, they are likely to correlate with injury severity. It is therefore not surprising that many of the mediators can correlate with outcome and this may be confounded by the severity of injury, rather than a specific facet of the biology of the mediator of interest.

This is further complicated by the fact that most studies focus on a small panel of mediators such as the “pro”-inflammatory mediators such as IL-1β, IL-6, TNF, and IFN-γ as well as “anti”-inflammatory cytokines IL-4 and IL-10. However, we should not discount the importance of other unmeasured mediators in contributing to the detrimental inflammatory responses after TBI and SAH. Cytokines are known to act in complex cascades with some early potent mediators that subsequently trigger the synthesis of regulatory cytokines which act to temper the initial response. Although in some cases cytokines have partially overlapping functions, each cytokine has a unique role, pattern of expression, and cellular source. Moreover, cytokines and chemokines display effects that are both “damaging” and “reparative” that may occur simultaneously in the post-injury phase (57). This dual effect has been also acknowledged for microglia and macrophages as both play both a beneficial and detrimental role in the injured brain (9, 14).

## Combination Therapy

Biological redundancy in complex interrelated systems means that there are multiple pathways to secondary neuronal injury (45). However, in most clinical trials, only a single therapy is used to target a single pathway limiting the potential efficacy. This is a potential reason why there is still no approved drug that has shown any clinical efficacy in mitigating neuronal injury in TBI and SAH. However, polytherapy using preclinically proven neuroprotective drugs (i.e., progesterone and Vitamin D hormone, creatinine, and choline) has also failed to show any treatment benefit (132), presumably as there are fundamental pathological differences between animal models and human brain injury (133) and also because we need a better understanding of the underlying pathophysiology and how the different pharmacological agents interact.

Nonetheless, a review of agents negating the neuroinflammatory cascades in brain injury indicates that there are many promising pharmaceutical agents currently being developed (43). Furthermore, with the tools currently available, it is increasingly feasible to monitor the neuroinflammatory response to therapy, thus allow researchers to rationalize specific combinations of therapy.

## Challenges Ahead

There are several challenges and limitations in the field today. The major issues surrounding these techniques discussed in this manuscript are summarized in Table 2.

**Table 2:** Limitations in current neuroinflammation literature.

Issues	Limitations	Suggested approaches
Biological compartments	Brain-ECF best for receptor and brain tissue biology, while CSF easier to collect and available in larger volume. Both feasible only short term. Blood is readily accessible for any injury severity, multiple sampling possible but less specific for brain pathology. Direct tissue sampling has the highest spatial resolution but difficult to acquire	Combining multiple samples, microdialysate has specific advantages for drug studies but should be combined with serum/CSF to reflect the global production of mediators

Monitoring time frame	Varying time-frames to insult, some neuroinflammatory cytokines have brief early temporal profiles and thus late monitoring may miss some biological signals	– Correct to time of injury – Beware false negatives – Late inflammatory monitoring (6–12 months) is associated with chronic sequelae

Collinearity and confounding	Several studies only measure a small number of mediators and are thus inferring causation incorrectly as other mediators may confound results	– Multivariate statistics are necessary – Need to measure multiple mediators simultaneously to avoid bias – Interventional studies required to infer causation

Regional vs global monitoring	Signal to noise ratio with dilution of mediators vs missing focal lesions. How representative is the data?	Combinatorial approaches, e.g., focal monitoring, global biomarkers, and neuroimaging

Microdialysis methodology	Protein microdialysis requires specific approaches to improve relative recovery	– Dextran or albumin should be used as carriers to increase relative recovery – Sensitive assays necessary as low concentrations are common – Multiplex technology allows simultaneous measurement of several cytokines

Clinical follow-up	Clinical outcome metrics such as Extended Glasgow Outcome Scale, SF36 may be insensitive and fail to capture subtle neurocognitive sequelae	Several modalities of outcome assessment necessary after as long as 12 months after ictus, including – Cognitive – Neuropsychological – Quality of life – Psychiatric – Functional outcomes

Neuroimaging	Difficulties in inferring what NMR, and TSPO PET binding represents at cellular level in relation to neuroinflammation	Combinatorial approaches necessary for future research, e.g., focal microdialysis monitoring plus neuroimaging

Systemic injury	Polytrauma might contribute to peripheral inflammatory response, which may modify or overlap with central neuroinflammatory response	– Accurate definition of patient population and injury assessment – Measurement of brain compartments vs extracranial components

Tissue outcomes	Difficult to access tissue samples unless associated with a surgical procedure	Tissue biopsies present a way to accurately describe the focal inflammatory response. Can complement other techniques

Autoimmune response	Empirical evidence that the adaptive immune system involved, but not clear if epiphenomenon or causative in inflicting neuronal injury	– Relating innate to adaptive immunity is developing field – Issue of cellular elements vs humoral

SAH neuroinflammation	Several pathological entities can overlap: early brain injury, vascular spasm, tissue ischemia each with its own neuroinflammatory signature	Careful characterization of the clinical state at the time of monitoring

Preclinical experiments	Molecular and cellular events that drive neuroinflammatory responses in the acute and chronic phases in traumatic brain injury requires animal or *in vitro* models for better bed-to-bench side translational research	– Systematic consideration of age, weight, species, sex to highlight variations in the neuroinflammatory response – Large animal models necessary to replicate human injury patterns (gyrencephalic brain, greater volumes for sampling etc.) – Improved outcome metrics that are adequate representations of human conditions – Better collaborations between clinicians and preclinical researchers to address the caveats in current research paradigms

*Table illustrating current issues, limitations, and suggested approaches*.

*MD, microdialysis; CSF, cerebrospinal fluid; SF36, short form questionnaire 36; NMR, nuclear magnetic resonance; TSPO, translocator protein; PET, positron emission tomography; SAH, subarachnoid hemorrhage*.

To measure and assess inflammatory activity, several current studies investigate a single marker in a single biological compartment. As discussed above, to adequately understand the neuroinflammatory response requires combinations of methodologies looking at multiple down-stream cytokines (59, 121), but also region specific focal inflammatory activity using PET-MRI (81, 82) and T-cell mediated autoreactivity toward brain-specific proteins (108).

Moreover, more sophisticated biostatistical methods analogous to the “-omic”-literature are required. Multivariate methods incorporating the entire cytokine profile using tools such as principal component analyses will presumably be necessary until we reach a greater understanding of these processes (57, 59).

In summary, we suggest a multimodal approach for the measurement of cytokines in various fluids, parallel to neuroimaging techniques to visualize changes in cellular/microglia activation with repeated measurement of both humoral and cellular immune activation to distinguish acute from chronic responses. Only this holistic approach will provide a deeper understanding of the longitudinal inflammatory processes in hope to develop accurately targeted and efficacious anti-inflammatory therapies.

## Conclusion

The neuroinflammatory cascade following TBI and SAH comprises a wide array of both humoral and cellular players. An ever-growing literature has defined changes in cytokine production and cell activation in both the acute and chronic phases following brain injury. However, we are still confronted with the difficulty in placing the individual components into a coherent picture. The extended time course of inflammation following these conditions provides multiple opportunities for therapeutic intervention. However, there is a limit to what we can learn from observational clinical studies as to the role of individual mediators/cells and ultimately understand how they affect patient functional outcome. For this reason, it is pivotal to maintain a close dialog between clinical and experimental research, in order to identify the distinct role of cytokines and immune cells, acting in the large scheme of the complex inflammatory responses.

## Author Contributions

ET, AH, TT, FZ, MM-K, DM, PH, and KC designed and planned the study. ET, AH, TT, FZ, MM-K, DM, PH, and KC drafted the manuscript, which all authors read and approved.

## Conflict of Interest Statement

The authors declare that the research was conducted in the absence of any commercial or financial relationships that could be construed as a potential conflict of interest.
